# Viewpoint on the Second Transatlantic GPCR Symposium for Early Career Investigators

**DOI:** 10.1021/acsptsci.2c00224

**Published:** 2022-12-16

**Authors:** John Janetzko, Cory P. Johnson, Paula Morales, Magdalena M. Scharf

**Affiliations:** † Department of Molecular and Cellular Physiology, Stanford University School of Medicine, Stanford, California 94305, United States; ‡ MDI Biological Laboratory, Bar Harbor, Maine 04609, United States; § Instituto de Química Médica, Consejo Superior de Investigaciones Científicas, Madrid 28006, Spain; ∥ Department of Physiology and Pharmacology, Karolinska Institutet, 171 77 Stockholm, Sweden

**Keywords:** G protein-coupled receptors (GPCRs), early career investigator (ECI), virtual symposium, career development, diversity and inclusion

## Abstract

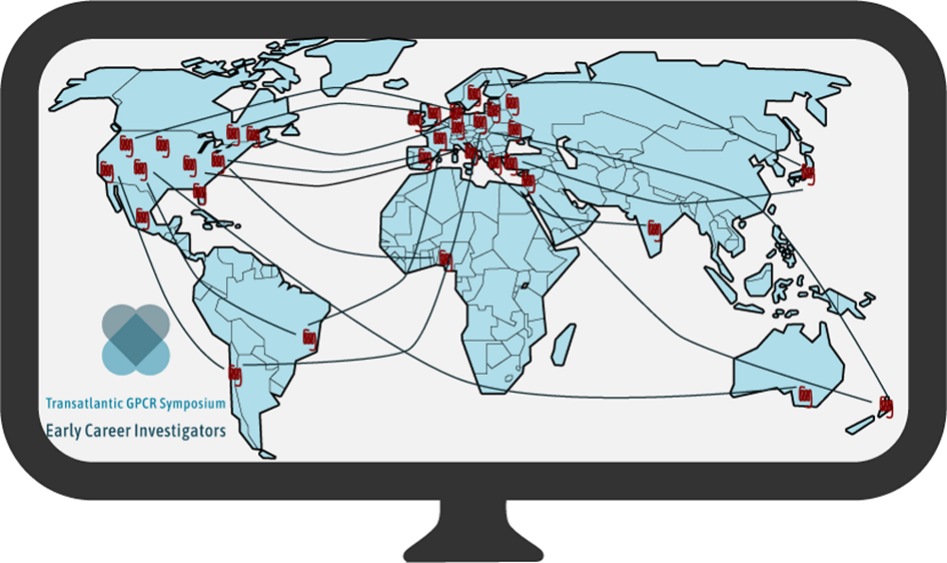

The second Transatlantic Early Career Investigator (ECI) G Protein-Coupled Receptor (GPCR) Symposium was an online scientific meeting geared at young GPCR investigators, with the primary goal of expanding opportunities for sharing research and networking among trainees in North America and Europe. Here, we discuss the format of our meeting, its impact, and the challenges and opportunities facing meetings like it in the future.

With in-person scientific meetings being canceled due to the COVID-19 global pandemic, virtual conferences served as a crucial means for presenting research and networking. This is particularly important for early career investigators (ECIs), for whom building their scientific networks is necessary for career advancement. In 2021, with support from The European Research Network on Signal Transduction (ERNEST),^1^ our predecessors Nicole Perry-Hauser, Brian Bender, Andreas Bock, and Desislava Nesheva launched the Transatlantic ECI GPCR Symposium as a means for early career researchers in North America and Europe working in the field of G protein-coupled receptors (GPCRs) to present their work and expand their professional network. Following the success of the first edition of this meeting,^2^ our goals as the new co-organizers for the second edition were (1) to maintain or expand the reach to a wide audience; (2) to improve on the format of the meeting; and (3) to expand opportunities for ECIs to present their work. Here we discuss the general structure of our meeting, its impact, and our views on the challenges and opportunities for similar meetings.

## Meeting Structure

The first edition of the meeting was a 10-hour one-day symposium. However, since time zones represented in a virtual meeting spanning North America and Europe range from Pacific Daylight Time (UTC–07:00) to Eastern European Daylight Time (UTC+03:00), the one-day meeting format posed challenges with scheduling, particularly if we wanted to expand it. To overcome this, we structured the second meeting as two 6-hour half-days, each with a career development element, talks, poster presentations, and networking opportunities. In doing so we achieved our goal of an expanded agenda without exacerbating the logistical challenges with timing. For career development/networking, our meeting included two informal “breakfast/lunch/dinner sessions” with faculty members—one with established “senior” faculty members and one with “junior” faculty members or those imminently launching their independent groups. We also included a career panel featuring six researchers who could speak to GPCR research opportunities in academia, industry, and biotech. Regarding the latter, we chose to focus our panelists on the discussion of research careers in different settings rather than alternative careers, given time constraints. Lastly, we provided an open networking space via Wonder (https://www.wonder.me/) where attendees could meet outside of the sessions to discuss collaborations, job openings, or anything else they were interested in. Our scientific program featured two keynote speakers, Miriam Stoeber (University of Geneva) and David Olson (University of California Davis), both of whom are young rising stars in studying GPCRs in neuroscience, as well as five oral presentation sessions (each 3 talks) and two poster sessions for ECIs to present their research. Notably, by having 15 oral presentation slots, this was a 50% increase over 2021.

## Impact and Demographics

**Figure 1:**
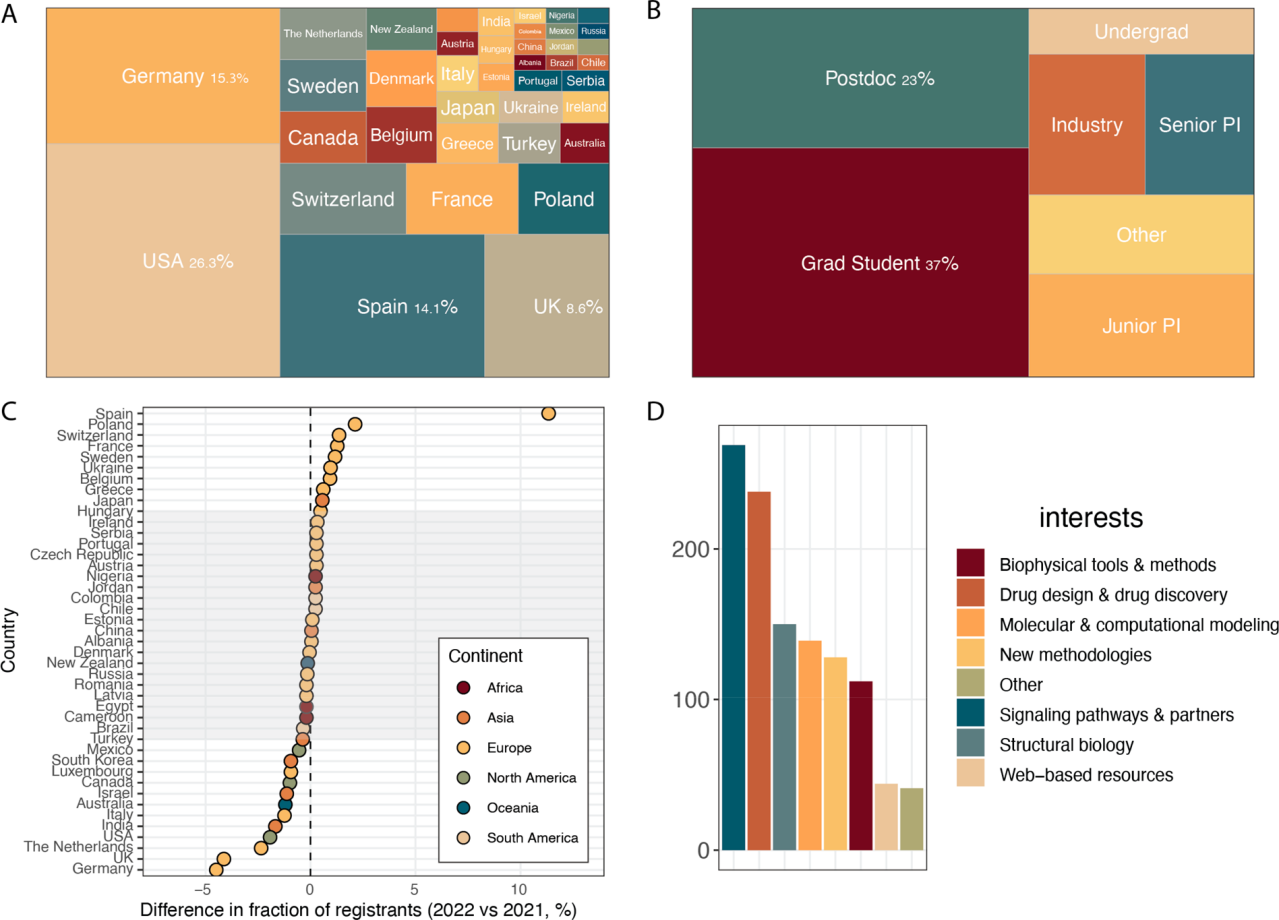
(A) Treemap showing countries represented at the 2022 symposium. Size of box is proportional to the fraction of whole. Percentages are shown for the most highly represented countries. (B) Treemap showing registrants by career stage. Size of box is proportional to the fraction of whole. Junior PI denotes a faculty member <10 years in an independent role. Grad student denotes both Ph.D. and master’s students. (C) A plot of the change in the fraction of registrants from a given country between the 2021 and 2022 meetings, colored by continent. The gray rectangle denotes a change of <0.5% between the two meetings. Negative changes indicate reduced registrations in the 2022 meeting. (D) Distribution of research interests for 2022 registrants. Registrants were asked to indicate at least one, and up to six interests from a multiple-choice list at time of registration.

Our symposium included people from 39 countries, across all continents, and had more than 400 registrants (Figure 1A). Surprisingly, even though the symposium was targeted to attendees in North America and Europe, still ∼5% of registrants were from countries including Japan, New Zealand, and Australia, for whom much of the symposium took place in the middle of the night. Compared to the 2021 meeting, we had fewer total registrations (419 compared to 520), even with more advanced notice and extensive advertising. However, these numbers still indicate high overall interest in the symposium. Comparing participation by career status (Figure 1B), 65% of registrants were early career investigators, with the largest fraction being graduate students [Ph.D. and masters] (37%), followed by postdoctoral fellows (23%) and undergraduate students (5%). Registrants from industry accounted for ∼8%, and faculty members/group leaders accounted for ∼19%. Overall, this breakdown by career stage aligned well with our goal of being an ECI-centric meeting; however, one opportunity for future growth is to expand the number of undergraduate student and research assistant attendees, which together accounted for ∼10% of registrants. Compared to 2021, we saw an increase in the proportion of registrants from Europe (64% in 2022 vs 55% in 2021), much of which can be attributed to the large increase in participation from Spain (Figure 1C). Countries which were less represented in 2022 by registrations were Germany and the UK; however, both countries were still among the top four countries represented at the meeting (see Figure 1A). Given the demographic shifts observed between 2021 and 2022, it is tempting to suggest that one driver of participation is having a co-organizer from that country. In 2021, Germany (Andreas Bock) and the UK (Desislava Nesheva) were both represented in the organizing team, whereas in 2022, Spain (Paula Morales) and Sweden (Magdalena Scharf) were represented, and both countries saw increased participation, while Germany and the UK saw reduced participation. This suggests that there is value in having co-organizers continue to represent different European countries, but that past organizers may need to remain involved in advertising and promotion. We note that in the symposium exit survey (72 responses, 18% response rate), about half of the respondents indicated that they attended the 2021 meeting. This also implies that our advertising campaign was effective at capturing a significant fraction of new participants. Finally, we surveyed the research interests of participants at the time of registration and found that while all research areas were captured, drug design and drug discovery were among the most common interests (see Figure 1D). While we aimed to make our agenda balanced across research topics, this suggests that there may be more opportunities to boost participation among those in industry (<10% in 2022) by expanding our coverage of this topic. Unfortunately, research interests were not recorded for registrants in 2021.

## Scientific Program Structure

Based on the research interests of our registrants, and topics we felt were pertinent to the current state of GPCR research we focused on five overarching themes for the meeting, including emerging tools, translational GPCR research, GPCR signaling, GPCR structure and biophysics, and computational approaches for GPCR research. We selected our two keynote speakers as individuals who not only were early in their independent careers but also captured the spirit that GPCR research today is increasingly multidisciplinary. David Olson and Miriam Stoeber’s research is at the interface of chemistry/neuropsychiatry and spatiotemporal dynamics/subcellular signaling, respectively. We purposefully selected one speaker to be from North America and the other from Europe, as well as one male and one female. Exit survey respondents expressed unanimous praise for both speakers. The remaining talks were intended to highlight exciting research being done by ECIs. We selected three oral presentations per topic, for a total of 15 talks from 35 submitted abstracts; everyone who submitted an abstract was encouraged to present a poster, and poster abstracts were accepted after the oral presentation deadline. While we selected speakers based on abstracts which we felt were the most exciting and well crafted, we also factored in a balance of geography, gender, and career stage. Notably, in the exit survey, most respondents believed that we achieved a good balance across all these areas. Our speakers included 2 undergraduate students, 8 graduate students, 3 postdoctoral fellows, and 2 research associates; they hailed from 8 different countries. In addition, between our two poster sessions we had more than 50 posters, bringing the represented countries to 16. Importantly, several attendees remarked that without this event being free and virtual they may not have been able to present their work.

## Highlights from ECI Research Presentations

While providing a platform for early career scientists to present their work, we also recognize that these scientists are the future of GPCR research. During the first session, new approaches to probe the biological and biochemical nature of GPCRs were highlighted. Aditya Kumar, a postdoctoral researcher from the University of Michigan, USA, spoke about their research using total internal reflection fluorescence microscopy to study the influence of membrane lipid order on endocytic pit formation. Next, Franziska Heydenreich, a postdoctoral researcher from the MRC Laboratory of Molecular Biology, UK, discussed the intersection of GPCR structure and pharmacological activity by characterizing the impact of each amino acid of the β2-adrenergic receptor on downstream signaling. Finally, Shivani Sachdev, a postdoctoral researcher at the National Institutes of Health, USA, discussed a novel technique using nanobody–ligand complexes to survey GPCR signal output.

The second session focused on new translational research. The first speaker, Louis Dwomoh, a postdoctoral researcher from the University of Glasgow, UK, presented his research indicating that the M1-muscarinic receptor activity provides a protective role in neurodegenerative disease. We then heard from Niklas Raven-Boess, a Ph.D. student from New York University, USA, who discussed the role of CD97 in glycolytic metabolism using patient-derived glioblastoma cells. Finally, Karim Ibrahim, a Ph.D. student from the University of Ottawa, Canada, described a deleterious role for the vesicular glutamate transporter 3 protein in a mouse model of Huntington’s disease.

The third session was dedicated to novel mechanisms in GPCR signaling. Gabriele Kockelkoren, a Ph.D. student from the University of Copenhagen, Denmark, explained how domains of membrane curvature–distinct from endocytic pits–influence the distribution and signaling of the β1-adrenergic receptor and other GPCRs. This was followed by Chloe Hicks, an undergraduate researcher at Duke University, USA, presenting on the biased signaling profiles of chemokine receptor CXCR3 in the presence of its three endogenous ligands. Lastly, Jenny Filor, a Ph.D. student at University Hospital Jena, Germany, discussed the critical role of the finger loop region in differential β-arrestin 2 recruitment to M2 and M4 receptors.

Session four featured computational tools and novel computational approaches in GPCR research. The first speaker, Claudia Llinas del Torrent, a Ph.D. student from Universitat Autónoma de Barcelona, Spain, described the molecular outcome of a point mutation in the adenosine 1 receptor found in patients with early onset Parkinson’s disease. This was followed by Marin Matic, a Ph.D. student at Scuola Normale Superiore, Italy, who developed an online tool for predicting GPCR-transducer coupling. Finally, Alessandro Nicoli, a Ph.D. student at the Technical University of Munich, Germany, presented a novel approach for modeling orthosteric binding sites in GPCRs with low-resolution structures using the example of an odorant receptor.

The fifth and final session of trainee talks elaborated on GPCR structures, conformations, and implications in GPCR signaling. Jiafei Mao, a postdoctoral researcher at Goethe University, Germany, explained how zinc ions act as allosteric modulators of the human bradykinin 1 receptor. Elise Bruguera, a Ph.D. student at Stanford University, USA, presented an approach to recapitulate purified heterodimers and oligomers in nanodiscs using split GFP. Lastly, William Steiner, an undergraduate researcher at the University of Utah, USA, described a novel G protein regulatory supercomplex with Smoothened in the hedgehog signaling pathway.

We believe our scientific program nicely illustrated two key points. First, young GPCR investigators are pursuing a broad range of exciting research questions involving GPCRs and doing so using a large repertoire of experimental methods. Second, excellent science is being done at all career stages. The latter point emphasizes the importance of giving researchers, especially those who are more junior, opportunities to present their work and the need for ECI-focused meetings such as this one.

## Career Development and Networking

In addition to scientific sessions, we included a career discussion panel and lunch sessions where trainees interacted with junior and senior GPCR group leaders in academia and industry. Our career panel included men and women from both academia and industry who provided a broad perspective to trainees about navigating their future careers. While this is not a unique session topic, our panel included researchers at both early and later stages in their career and incorporated entrepreneurial aspects of industry that we felt were unique to those working in the GPCR field. Complementary to our career discussion panel, we were able to provide attendees the opportunity to have an informal lunch conversation with both GPCR research legends, as well as up and coming leaders in the GPCR field. An unexpected consequence of our meeting was the creation of a junior PI meeting, with the goal of giving young PIs a new opportunity to meet, share ideas and build collaborations. This goes to show that online events like this one can provide low-barrier opportunities for community building.

## Key Challenges and Opportunities to Consider for Online Meetings

Planning a meeting is never easy; however, there are unique challenges specific to virtual meetings. As we believe there is tremendous value for meetings like this one, even as in-person meetings resume, we wish to bring these challenges to the attention of the community, particularly those who have not organized a virtual meeting.

### Platform Selection Is Important

It should be low cost (especially if the meeting is meant to be free to attend), able to handle the anticipated number of participants, easy to access and navigate between sessions, easy to use without further preparation, and ideally without the need to create an account.

### Online Conferences Are Not Free to Operate

There is a common misconception that organizing conferences online comes with little or no cost due to the lack of physical event space, meals, etc. While they are cheaper than their in-person counterparts, platform fees, website costs, technical support staff, and other expenses all add up. It is important for sponsors and funders to know that for these meetings to be successful and run smoothly a financial investment is necessary.

### It Is Difficult to Match the Interactivity of In-Person Meetings with Online Meetings

There is something to be said about conversations that develop organically at an in-person meeting, which has been challenging to replicate in online settings. Given that attendees do not see one another at a virtual meeting, there is often a sense of reduced interactivity, as was also mentioned by participants in the exit survey. Further, in our experience, networking sessions are unfortunately often perceived as “skippable” during a meeting, especially if the timing is inconvenient or if insufficient time is allocated to breaks. Improvements to the attendee experience, by enhancing their sense of connection as well as giving priority time to networking opportunities, will be critical for online meetings, especially those focused around ECIs. While improvements in technology, such as use of environmental exploration platforms (e.g., Wonder), have helped make communication easy, there remains more to be done to ensure that conversations occur more naturally (e.g., without lag) and result in lasting interpersonal connections and that attendees are able to interact more similarly to how they would in-person meetings.

### Timing of the Meeting Schedule Needs to Be Carefully Considered

Challenges that in-person conferences do not contend with are time zones and people’s limited tolerance for screen time. Unfortunately, these must be carefully considered when hundreds convene online across time zones spanning as much as a 10-hour difference in the most extreme case. This means that it is difficult to actively include an audience from all parts of the world, and even with careful considerations some attendees may find themselves attending sessions at somewhat inconvenient times. Furthermore, the time that can be dedicated to sessions during a virtual symposium is limited also by the fact that most people have a limited capacity for engaging with a computer screen. To make it easier for people to stay focused in front of their screen, it is helpful to have interactive sessions and breaks between talk-focused scientific sessions.

### It Is Difficult to Estimate Attendance Beforehand

Virtual meetings have a very low barrier for entry and almost no limit to the number of participants. While scalability is also a strength of online meetings, this results in the registration of many people who will not actually participate in all sessions or who may choose to attend only a subset of sessions. According to the exit survey, only 8% of respondents attended all sessions. The most common reasons for not attending a session cited by survey respondents were a prior commitment (54%), inconvenient timing (46%), or needed a break (31%). Importantly, only 14% indicated a lack of scientific interest was their reason for not attending a session. In this way, an online meeting is a double-edged sword: it is easy to squeeze into your normal work schedule without traveling or having to remove yourself from ongoing work in the lab; however, this also means that people often do not dedicate their full attention to a virtual meeting as they would to an in-person meeting. It is worth considering why we value a meeting we travel to more highly than one we can access from our computer, and whether this can be adjusted if online meetings remain ubiquitous in our lives.

### When Given a Choice We Prioritize In-Person Meetings over Online Meetings

From our own personal experiences, as well as anecdotes from others, it seems to be the case that if someone has a choice to attend *either* an in-person meeting or an online meeting, they will choose the in-person meeting more often. However, we emphasize that the community must not a priori regard an online meeting as “lesser” than an in-person meeting. We speculate that one of the causes for the reduced participation from many wealthy North American and European countries is the resumption of in-person meetings and people not seeing meetings like this one as a priority when they have access to in-person meetings. Yet, for many the constraints of traveling to an in-person meeting, whether it be cost, illness, childcare, or family obligations, make online meetings an important door to networking and scientific discourse for these individuals. We encourage the reader to choose an online meeting to prioritize similarly as an in-person meeting and to take full advantage of all aspects of the meeting, including any networking sessions. This will ensure that meetings like this one remain high-quality scientific forums where those who may not have the privilege to attend an in-person meeting can also maximally benefit.

Having discussed some of the challenges meetings such as this one face, we want to end by discussing some strengths of online meetings, and their position in the future. Indeed, 97% of survey respondents think that virtual meetings such as this one will have value even after in-person meetings are back. Virtual meetings create an equality that in-person meetings fail to achieve, especially if the online meeting is free of charge. This is especially helpful for ECIs and researchers from laboratories with tighter budgets who cannot afford the cost of attending an in-person international meeting. Further, online meetings do not suffer from borders: there are no visa/immigration issues. Finally, virtual meetings are a more environmentally conscious solution to connect scientists from around world.

## Conclusions

Overall, we believe that the second Transatlantic ECI GPCR Symposium was an extremely successful follow-up to the first edition of this meeting, with 99% of survey respondents indicating that they would like to see the symposium happen again. Still, given our experience and the feedback we received informally and through our exit survey, we believe this meeting can still be improved upon. The extension from a 1-day to a 2-day meeting made it possible to expand both the scientific and career development agendas while also having shorter days and more breaks. We are excited to share that the Transatlantic ECI GPCR Symposium is scheduled for a third edition in summer 2023 and will be co-chaired by Franziska M. Heydenreich (MRC Laboratory of Molecular Biology), Gabriele Kockelkoren (University of Copenhagen), Aditya Kumar (University of Michigan), and Sreeparna Majumdar (University of North Carolina Chapel Hill), and we look forward to seeing how the meeting evolves under their direction. Our experience organizing this meeting showed us the value of bringing together a diverse set of ECIs who may not otherwise have the same opportunities to network and present their research, and we hope that it and meetings like it will continue to be supported by their respective research communities and fill a niche distinct from the many traditional in-person meetings.

## Abbreviations

GPCR: G protein-coupled receptor
ECI: early career investigator
